# Longevity and germination of *Juniperus communis* L. pollen after storage

**DOI:** 10.1038/s41598-021-90942-9

**Published:** 2021-06-17

**Authors:** Andrej Kormuťák, Peter Bolecek, Martin Galgóci, Dušan Gömöry

**Affiliations:** 1 Plant Science and Biodiversity Center, Institute of Plant Genetics and Biotechnology, Slovak Academy of Sciences, Akademická 2, P.O. Box 39A, 950 07 Nitra, Slovak Republic; 2 Faculty of Natural Sciences, Constantine Philosophy University in Nitra, A. Hlinku 1, 949 74 Nitra, Slovak Republic; 3 Faculty of Forestry, Technical University in Zvolen, T. G. Masaryka 24, 960 53 Zvolen, Slovak Republic

**Keywords:** Genetic hybridization, Ecological genetics, Plant sciences

## Abstract

Pollen storage belongs among the most important activities associated with pollen handling. It overcomes the differences in pollen shedding and ovule receptivity during controlled pollination experiments. It is especially important for species like common juniper (*Juniperus communis* L.) with an extremely low quality of seeds due to pollination failure. Additionally, it is a substantial part of germplasm preservation programmes in pollen banks. In the present paper, the effect of short-term storage of pollen was studied using pollen samples from five shrubs in an in vitro germination test. Two temperature regimes were tested. The pollen viability of freshly collected pollen varied considerably between individual shrubs, exhibiting 67.3–88.6% germination rate and 248.0–367.3 µm of pollen tubes. Storage at + 4 °C for four months was accompanied by a profound decline in pollen viability. The germination percentage was reduced to 49.2–75.2% and the pollen tube length to 32.5–69.0%, depending on individual shrubs. The corresponding decline in pollen viability characteristics during storage at − 20 °C was only negligible in two of the tested shrubs. In the remaining three shrub samples, an increase in germination percentage was observed. Pollen tube growth responded more sensitively to freezing, but, on average, the decrease in length was lower than that at + 4 °C. The rate of reduction in pollen tube length varied between 11.5 and 45.4%. Cytological events accompanying in vitro germination of freezer-stored pollen exhibited some delay in releasing the exine from pollen grains during the early stages of germination as compared with freshly collected pollen. In conclusion, short-term storage of the common juniper pollen in a freezer is better for the preservation of its viability than storage at + 4 °C.

## Introduction

Common juniper (*Juniperus communis* L.) is an obligatory dioecious species of the family Cupressaceae with a wide distribution in the Northern Hemisphere. It may be found in North America, Europe, Asia, and in some areas of North Africa^1^. In Europe, this species extends from southern Spain to the arctic circle in Norway^2^. As a species with a broad ecological amplitude, the common juniper is spread across all of Slovakia with an altitude range extending from the lowlands up to 1495 m above sea level (a.s.l.)^3,4^. The common juniper is an anemophilous species, but a certain degree of entomophily is also quite common^5^. The transformation from entomo- to anemophily during the evolution of pollination mechanisms in gymnosperms has left some traces in the structure and function of ovules and pollen grains in conifers^6^. A distinctive feature of common juniper pollen grains is the mechanism of their germination, including the mechanism of its initiation. Hydration, as a prerequisite for metabolic activation of pollen grains and subsequent pollen tube formation, is accompanied by shedding of the exine, which is a phenomenon unique to gymnosperms^7^. After restructuring, pollen grains begin to germinate when in culture. Cytologically, the initial stages of the process share little analogy with other species of gymnosperms. Additionally, the duration of pollen germination on artificial media differs profoundly between *J. communis* of the family Cupressaceae and conifers of the family Pinaceae. The pollen grains of the Cupressaceae start to germinate in 4–5 days, and the pollen of Pinaceae needs 24–48 h to germinate with the pollen tubes reaching full growth within 3–4 days^8^. The other deviating feature that discriminates between species of these families is the potential of their pollen for long-term storage. Mature pollen of the Pinaceae dehydrates to less than 10% water content before being shed, resulting in remarkable aerial buoyancy. This allows the dehydrated pollen to be collected and stored at low temperatures (< − 20 °C) for several years. In contrast, mature pollen of the Cupressaceae has about 30% water content, making it difficult to store for a long period of time^9^. Unfortunately, this aspect of pollen biology has been neglected in the entire family. No study has appeared yet referring to the behaviour of pollen under storage. Preference has been given to pollen characteristics, such as the quantitative aspects of pollen production in *Olea europaea*^10^, pollen morphology and pollen surface structure in some *Juniperus* species^11^, and aerobiological and allergenic properties of the cypress pollen^12^. In common juniper, few studies have reported on the cytological mechanism of in vitro pollen germination^7,13^. Although common juniper is generally not regarded as a commercially important species, its fruits are broadly used in culinary and pharmacy. As such, it may become an object of breeding^14,15^. This may require artificial crossing, for which efficient pollen storage is indispensable. In the present paper, the effect of storage on pollen viability under two temperature regimes was studied along with the dynamics of cytological events accompanying in vitro germination of freezer-stored juniper pollen grains to obtain the first data of the kind in the common juniper.

## Results

**Figure 1:**
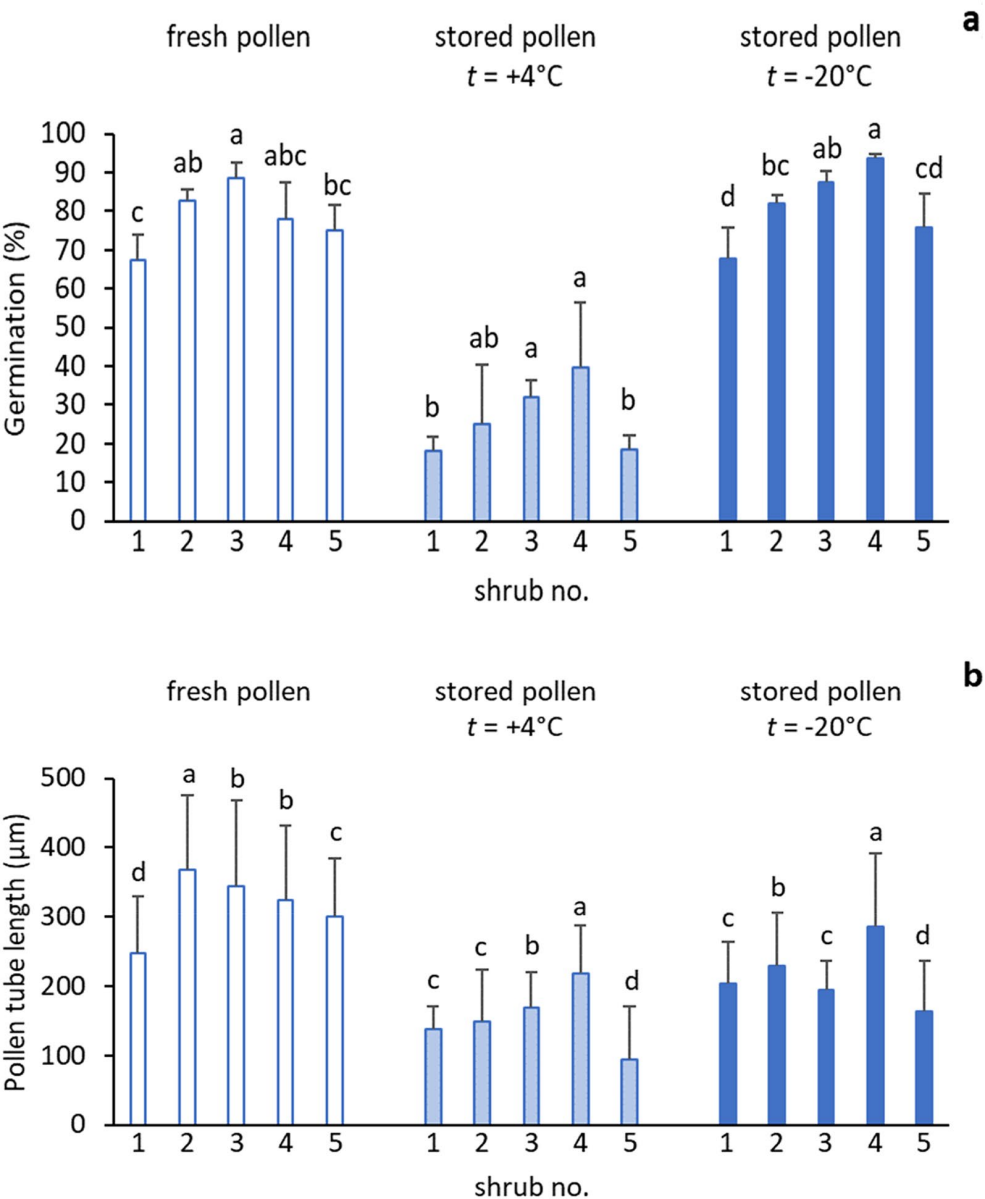
Graphical illustrations of variation in pollen germination percentage (**a**) and pollen tube length (**b**) of individual shrubs revealed in fresh pollen and in pollen under storage. Different letters refer to the statistical significance of the differences between tested individuals and storage variants, resulting from Duncan's pairwise tests.

A uniform response of the pollen grains towards storage conditions was registered in all five shrubs investigated with a conspicuous decline in germination percentage and pollen tube length after storage. Pollen tube growth reacted more sensitively to storage than germination. The most profound reductions in pollen viability traits were observed in samples stored at + 4 °C. The germination percentage of freshly collected pollen of individual shrubs ranged between 67.3 and 88.6%, whereas that in stored pollen was between 18.0 and 39.6%. In relative terms, storage represented a 49.3–73.2% decline in germination (Fig. 1). The same tendency was also observed in pollen tube growth, when freshly collected pollen possessed 248.0–367.3 µm long pollen tubes, and pollen stored at + 4 °C was characterised by 93.9–218.5 µm long pollen tubes. The corresponding decline reached 32.5–68.7%.

**Figure 2:**
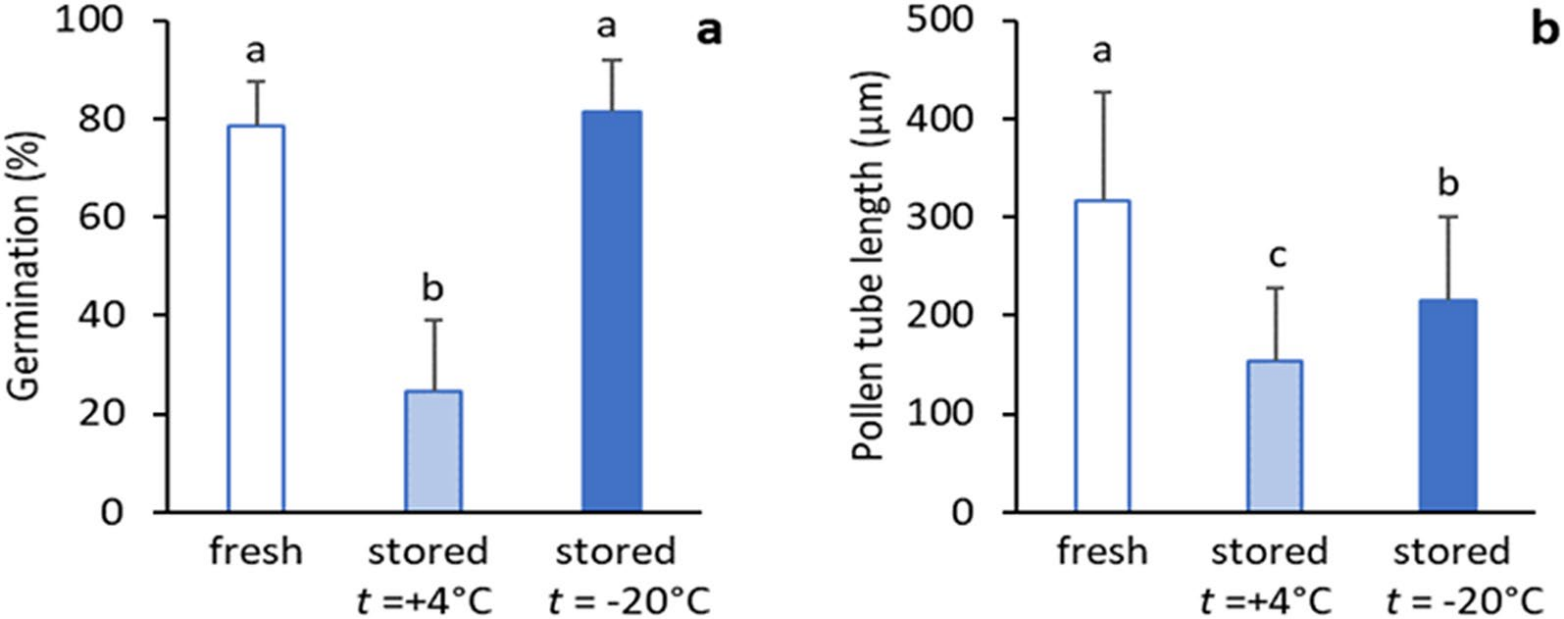
Average values of pollen germination percentage (**a**) and pollen tube length (**b**) of five shrubs of each fresh pollen and pollen stored at + 4 °C and − 20 °C. Different letters refer to the statistical significance of the differences between three groups of tested pollen, as revealed by Duncan's pairwise tests.

Contrary to storage at + 4 °C, pollen stored at − 20 °C had an increased germination by 0.3% in shrub no. 1 and 0.6% in shrub no. 5 as compared with fresh pollen. A more conspicuous increase in pollen germinability was registered in individual no. 4, exhibiting 70.0% germination in fresh pollen and 93.6% in pollen stored at − 20 °C. In the remaining two shrubs (no. 2, 3), only a negligible decline in pollen germination was recorded. The deviation from freshly collected pollen varied within 0.5–16.8%. In general, the germination characteristics of pollen stored at − 20 °C were comparable with those of the fresh pollen and varied between 67.6 and 93.6%. As a second viability trait, pollen tube growth deviated more profoundly from that of fresh pollen than germination. On average, the pollen tube length of pollen stored at − 20 °C ranged from 163.0 to 286.6 µm, which represents a 11.4–45.7% decline compared to fresh pollen (Figs. 1, S1). ANOVA and Duncan`s grouping confirmed the highly significant differences between tested shrubs in both pollen germination percentage (*P* < 0.019) and pollen tube length (*P* < 0.0001) of the freshly collected pollen and the two storage conditions. Summarily, the pollen viability data of these conditions is illustrated in Fig. 2.

Based on Duncan`s test, the freshly collected pollen and pollen under − 20 °C storage had a comparable germination percentage. Statistically significant differences between the three pollen samples were due to the low quality of pollen stored at + 4 °C. Pollen tube growth was more sensitive to storage temperature as evidenced by a profound difference in pollen tube length under the tested storage conditions, which was confirmed by the ANOVA test (Analysis of variance). It follows from the data presented in Table 1 that except for the interaction between storage temperature and individual shrubs, the differences in pollen viability traits were highly significant between storage temperature variants, individual tested shrubs, repetitions, storage temperature, and tested individuals.

**Table 1:** Analysis of variance of juniper pollen germinability and pollen tube length in fresh and stored pollen.

Source	DF	Germination percentage			Pollen tube length		
		Mean square	F	*P*	Mean square	F	*P*
Temperature	2	1.883998	216.35	0.0001	3,048,232.807	711.94	0.0001
Individuals	4	0.115089	13.22	0.0001	377,142.883	88.08	0.0001
Temp × individual	8	0.017327	1.99	0.0826	96,261.348	22.48	0.0001
Repet (temp × ind)	30				110,091.447	25.71	0.0001
Error		0.008708			4281.59		

**Figure 3:**
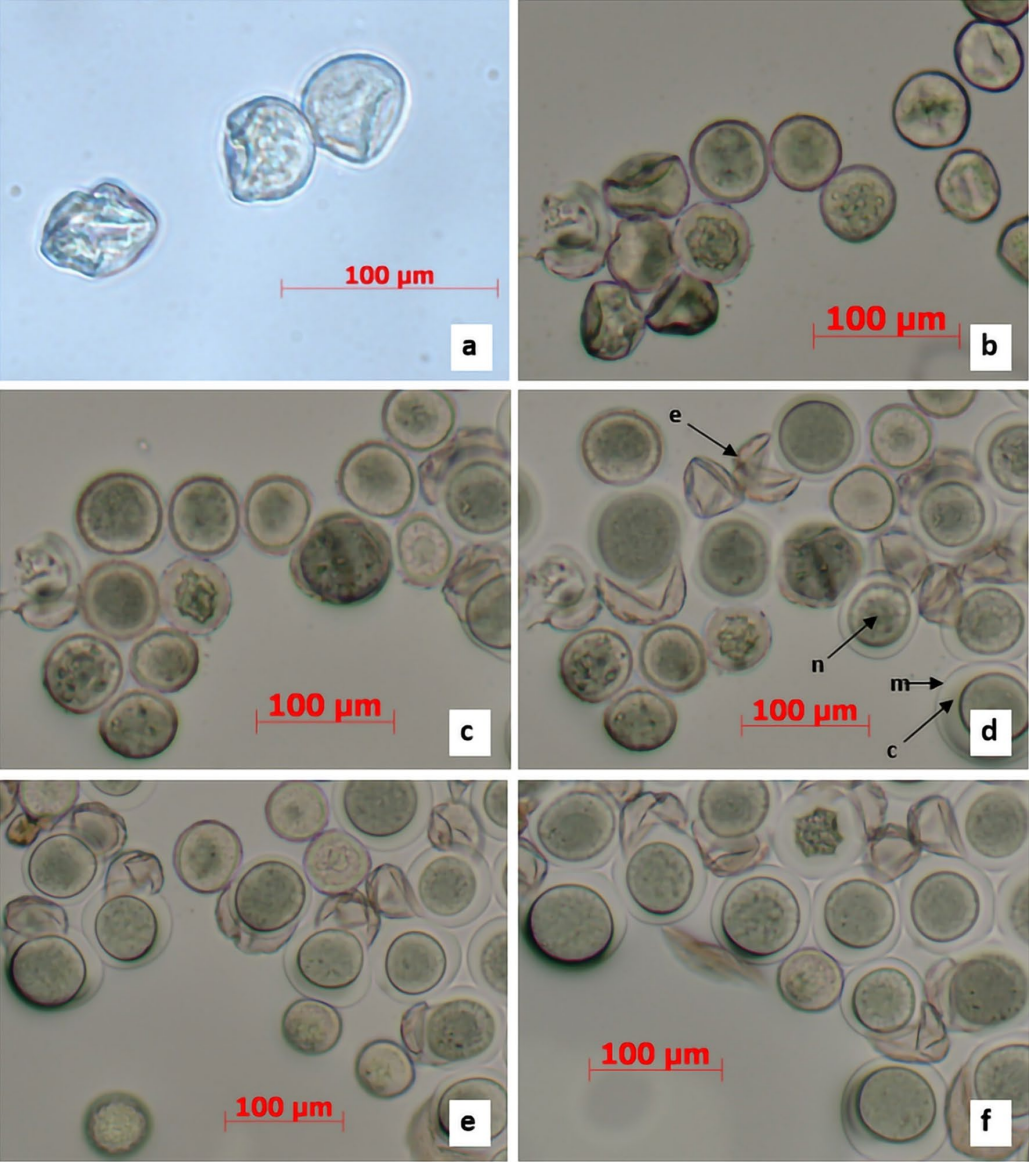
Dynamics of pollen hydrophilic capsule formation in pollen grains immediately after sowing on media (**a**) and subsequent changes after 10 (**b**), 50 (**c**), 55 (**d**), 60 (**e**), and 90 min (**f**) of cultivation; e-exine, m -hydrophilic capsule membrane, n-pollen nucleus, c-hydrophilic capsule. The photos were taken using Axioplan 2 microscope with built in AxioVision 4 software (Carl Zeiss Vision Gmb H).

Cytological investigation of the pollen germination mechanism in freezer-stored samples indicated some deviations from the process involved in fresh pollen. Immediately after transfer from the storage container to cultivation media, the dormant pollen grains appeared as contracted structures of spherical and rectangular shapes with a conspicuously structuralised exine surface (Fig. 3a).

**Figure 4:**
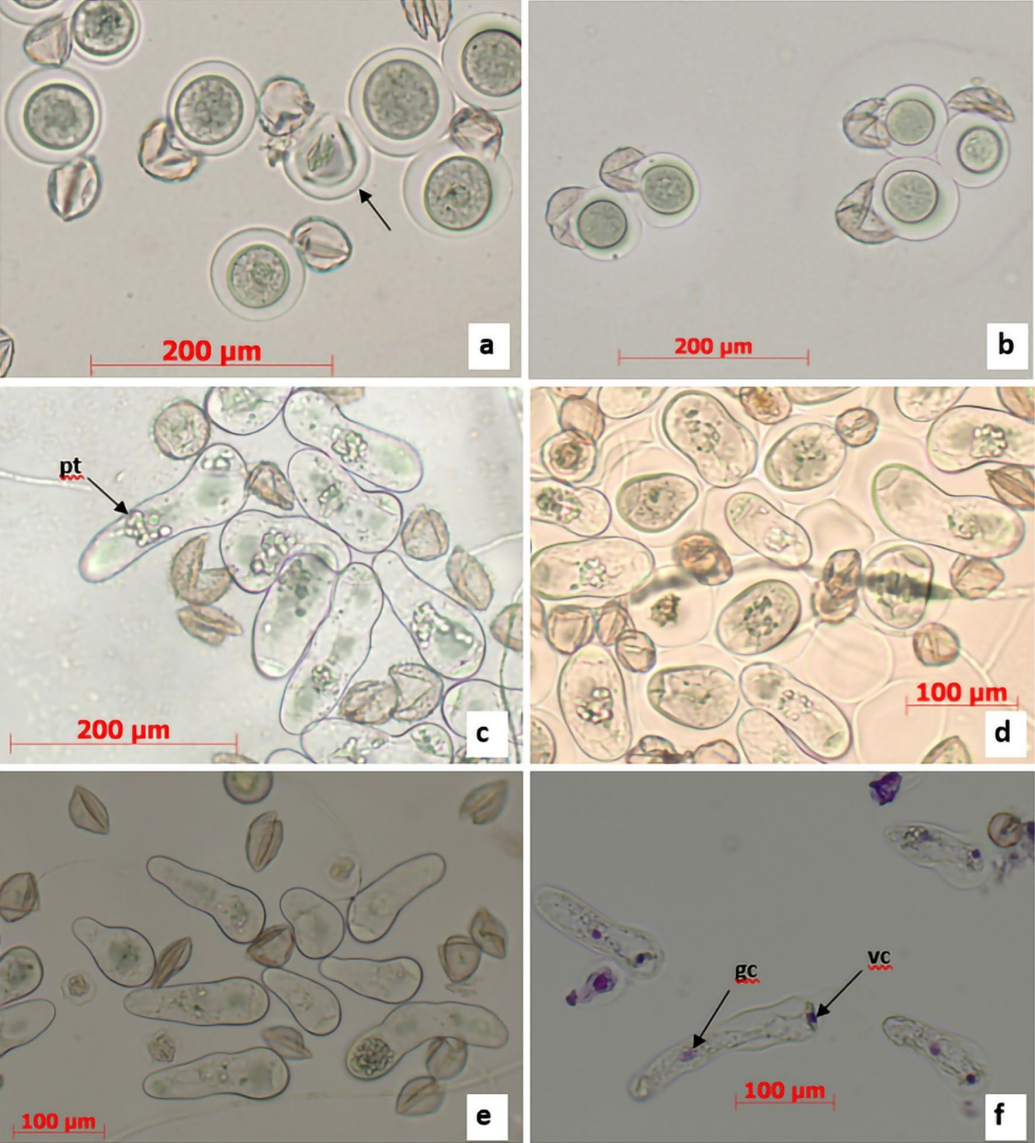
Dynamics of pollen germination showing pollen tube initiation (**a**), persisting hydrophilic capsules of pollen (**b**), and early (**c**,**d**) and advanced stages of pollen germination (**e**,**f**); *pt* pollen tube, *gc* generative cell, *vc* vegetative cell.

The sequence of events accompanying the early stages of pollen germination were cytologically followed for 90 min after plating. Ten minutes after sowing pollen on the nutrient media, the prevailing amount of pollen grains remaining in a shrivelled stage indicated delayed imbibition (Fig. 3b). A massive detaching of the exine from pollen grains took place 50–55 min after pollen sowing (Fig. 3c,d). This unique phenomenon was caused by pollen hydration due to intense water penetration through the functional pore of the exine. The mucilaginous substance of the intine, containing abundant polysaccharides, swelled and caused the exine to burst, which was left on the media in the form of two half-opened valves with their edges curved inwards^13^. In some pollen grains, the exine had not yet been detached from the pollen body at this stage of pollen imbibition (Fig. 3c,d). As a result of continuing hydration, the hydrophilic capsule of the pollen grains became rather distinct, enlarging its volume during the 60–90 min period of pollen incubation. Externally, the capsule was bordered by a semipermeable membrane of the endoexine and internally by the intine (Fig. 3d–f). After 2–3 days of cultivation, the intine began to elongate, giving rise to the pollen tube. The entire process of pollen tube formation took place within a hydrophilic capsule and was rather asynchronous among pollen grains (Fig. 4a,b). The asynchronous

nature of pollen tube growth became more apparent after 5–6 days of pollen cultivation on the media, when more advanced stages of the process were observed, involving an elongated intine, shoe-like pollen tubes characteristic of the early stages of pollen tube growth, and elongated linear pollen tubes (Fig. 4c,d). Pollen germination took place on the background of contaminated nutrient media with numerous bacteria and fungi spreading over the surface. The germination potential of pollen grains was fully expressed on the seventh day of cultivation. The viable pollen grains with elongated pollen tubes appeared as very compact structures (Fig. 4e). Feulgen staining revealed that the haploid nucleus of a microspore had divided, giving rise to the pollen tube and vegetative cell (Fig. 4f).

## Discussion

Short- and long-term storage are among the most important activities associated with pollen handling. It enables breeders to overcome differences in pollen shedding and ovule receptivity during controlled pollination of trees on the same locality or trees in widely separated regions^16^. Storage temperature, moisture content of the pollen, and relative humidity during storage play a decisive role in retaining the viability of stored pollen^17^. Optimal values of these factors and their combination may differ in individual groups of plants, depending on the structure and chemistry of pollen. In the present study, the effort was made to standardise the relative humidity within desiccators used for + 4 °C and − 20 °C storage. In both containers, the relative humidity was maintained at 27–30%, approaching the humidity level suggested for pollen storage in conifers (10–22%)^18,19^. Moisture content of the pollen was not determined during the study, but all five pollen lots sampled were uniformly dried under laboratory temperatures before storage. As for the temperature variable, the storage temperature for pollen with a high moisture content has been recommended to be around 0°C^20^. Conversely, our results showed unequivocally that storage at − 20 °C was a better alternative for the storage of juniper pollen. This conclusion was supported by a profound decline in pollen viability parameters in pollen stored at + 4 °C compared with the viability of fresh pollen. In contrast, only a slight decline in pollen viability after storage at − 20 °C was observed, and in some samples, a stimulatory effect of this temperature on pollen germination occurred. This may be taken as confirmation that temperatures around − 18 °C and − 20 °C, which are recommended for the storage of pine pollen^9,21^, are equally suitable for storage of *J. communis* pollen. Although there are several reports on the complete retention of pollen germination and pollen tube length in pine (*Pinus mugo*) pollen after one year of storage in the refrigerator^22^ or of a negligible decrease in germination in *Pinus radiata* and *P. banksiana* after one or two years of storage at + 4 °C^8,23^, the response of juniper pollen to short-term storage under similar temperatures was much more severe. This applies primarily to pollen tube growth, while the effect of cold storage on germination rate was less pronounced, especially at − 20 °C. Apparently, even when the capacity of stored pollen grains to germinate was less affected compared to fresh pollen, recovery from low temperatures may have partly exhausted the energetic resources of a pollen grain, resulting in a certain loss of vigour. The apertures on the exine have a high hydrophilicity that explains the death of pollen when it is stored for a few months at > 10% relative humidity^13^. Obviously, further research is needed to elucidate the conditions necessary for juniper pollen germination to occur after a longer period of storage. The approach based on in vitro germination shed some light on the germination mechanism, contributing simultaneously to characterisation of the pollen viability potential in quantitative terms. The ascertained germination of freshly harvested juniper pollen ranged between 67.3 and 88.6%, which was lower than that of fresh pollen in *Pinus sylvestris*, *P. mugo,* and their hybrids (88.2–95.4%)^24^. In a study on the effect of nitrogen fertilisation on the quality of *J. communis* pollen, a relatively low germination rate in pollen collected from fertilised plants (1.36%) was reported compared with non-fertilised plants (24.9%)^25^. The low pollen quality was considered one of the factors responsible for reproductive failure in natural populations of common juniper. A direct transfer of freezer-stored pollen onto a nutrient media surface, without previous preconditioning, adversely affected some processes associated with in vitro germination. During the taxoid type of pollen hydration, the exine is released from a pollen grain within 3–4 min.^7^. In our study, the entire process was completed within 60–90 min, during which an overwhelming majority of the pollen grains formed their hydrophilic capsules. The process was originally reported to take place in both viable and dead pollen grains, but recent data indicated that only 75% of incubated pollen grains released their exines after 96 h of incubation^25^. This is an additional reason for the continuation of pollen germination experiments aimed at elucidating aspects of the pollen germination mechanism in common juniper.

Summarily, the presented results on pollen storage in common juniper may be taken as preliminary studies because of the limited period of storage. Nevertheless, the experiment showed a higher sensitivity of juniper pollen to storage at + 4 °C compared, for example, to pine pollen. Conversely, freezer-storage at − 20 °C seemed more promising for retaining pollen viability, as a prerequisite for overcoming differences in the receptivity of juniper reproductive organs over different seasons. This is a necessary step in carrying out artificial pollination experiments with juniper oriented toward improving the poor quality of seeds. The latter is believed to be caused by reproductive failure in natural populations, causing the species to become endangered^26^. Only a 1.94% yield of fully developed seeds was postulated for juniper under natural conditions^26^, which is a big challenge to a better understanding of the events involved in the species’ reproductive cycle. In a broader sense, seed abortion was ascribed to pollination failure and to the factors related to physiology and nutrition of the female shrubs during the long maturation of the cones^26^. However, a more detailed specification of the factors responsible for this phenomenon was not provided. Therefore, it is reasonable to expect that intensive study on pollen quality, dispersion of pollen during the flowering period, and efficient pollination of the ovules, which ensures undisturbed embryogeny, might shed more light on the intricate mechanism of sexual reproduction in *J. communis*.

## Conclusions

The presented results on longevity of stored common juniper pollen may be taken as a preliminary study. Nevertheless, the experiments demonstrated the higher sensitivity of juniper pollen to storage at + 4 °C compared, for example, to pine pollen. Conversely, freezer-storage at − 20 °C seemed more promising for retaining pollen viability, as a prerequisite for carrying out a supplementary pollination of the species to improve the quality of its seeds. The latter is believed to be caused by reproductive failure in natural populations, causing the species to become endangered. Regarding a critically endangered status of several *Juniperus* species around the world and expected similarity in pollen properties, the presented data may be of interest also for the conservation programmes, especially in establishing the pollen banks of the species.

## Materials and methods

### Plant material and short-term storage conditions

Male shrubs of the common juniper (*Juniperus communis* L.) growing in Dubniky of Western Slovakia (340 m a.s.l.) were used for study. The locality is situated near the village Bukova on a foot of the Little Carpatian Mountain Ridge and represents a public pasture land with advanced secondary succession. As common juniper is not a protected species in Slovakia, and the locality represents public land, collection of material for research does not require any specific permission, and was done in accordance with the national legislation (Act No. 543/2002 Z. z. on conservation of nature and landscape, §§ 4 and 47, Act No. 220/2004 Z. z. on protection and use of agricultural land). Twigs with mature microstrobili were cut separately from 5 shrubs and transferred to vessels with water and taken to the laboratory 2 days before discharging the pollen from microsporangia. Released pollen grains were collected on a large sheet beneath the vessels and sieved. The pollen grains of each individual were, after sieving, left in the laboratory for one additional day to dry more completely and used immediately in a germination test. This portion of samples was attributed to fresh pollen. The remaining dried pollen was divided into two parts in each tested shrub. One part of the pollen was stored at + 4 °C, the other at − 20 °C. The pollen was stored in glass test tubes that were plugged loosely with cotton-wool caps and placed into a desiccator with calcium chloride. The two temperature regimes included refrigerator storage at + 4 °C and freeze-storage at − 20 °C. The relative humidity inside the desiccators was 27–30%. The pollen was stored for a period of four months.

### In vitro germination

Pollen germination was carried out at 25 °C in the dark with a separate evaluation of pollen viability in each shrub under study. The cultivation media consisted of 1.5% agar and 5% sucrose^27^; the latter has been reported to be optimal for common juniper pollen germination in culture^5,7^. The pollen was evenly dusted over the gel surface in small Petri dishes (47 mm diameter) by blowing it from a soft-hair brush. Each dish was placed in a larger Petri dish (70 mm diameter) containing 2 ml H_2_O and covered with a lid. Each sample was tested in triplicate. Immediately after sowing, the dynamics of pollen hydrophilic capsule formation was followed by 10, 50, 55, 60, and 90 min cultivation. Plates were incubated for 7 days, which was necessary for juniper pollen to fully express the growth potential of pollen tubes^5^. During the second half of the culture period, contamination of the cultivation media occurred by fungal spores and bacteria on the pollen surface and from the air^8^. The number of germinated pollen grains was recorded from a random sample of 100 pollen grains, and the pollen tube length was measured on a sample of 30 pollen grains on each Petri dish (total of 300 pollen grains and 90 pollen tubes per shrub). The evaluation was made with a NU 2 microscope (Carl Zeiss Jena) using a 10× eyepiece with a scale and 25× objective. A pollen grain was considered viable when its pollen tube reached a length comparable to the diameter of the pollen grain body^28^. The presence of clearly differentiated pollen tubes in individual pollen grains served as a main criterion of pollen viability. Another viability criterion was mitotic division of the microspore nucleus, giving rise to the vegetative and generative cells, both of which were visualised in growing pollen tubes by Feulgen staining. When measuring the length of a pollen tube, the distance from a zone where the round shape of the pollen grain body extended into the pollen tube was considered to the tip of the pollen tube. In total, the germination percentage evaluation was based on 3000 counts involving three repetitions per individual and 5 shrubs with freshly collected and stored pollen. Pollen from each of the shrubs was evaluated separately. Likewise, the pollen tube length data included 900 measurements. Differences in both viability traits were tested regarding the storage temperature and shrubs using two-way analysis of variance (ANOVA). Germination percentages were arcsine transformed prior to analysis. Calculations were performed using the GLM (General linear model) procedure in the statistical package SAS, version 9.4. Pairwise differences between individual shrubs were tested by Duncan`s test.

### Feulgen staining of microspore nuclei

Germinating pollen grains were washed out from the cultivation medium surface with 1 N HCl into a thin-walled glass test tube using a pipette. The test tube containing pollen was plugged with a rubber stopper and incubated in a water bath (+ 60 °C) for 5 min. The hydrochloric acid was subsequently drained carefully from each tube. Feulgen solution was added to the pollen grains stuck on the tube wall. The test tube was plugged with a stopper and left at room temperature in the dark for 1 h. In the same way as with hydrochloric acid, Feulgen solution was discharged from the tube to which 7% acetic acid was added. A few drops of the mixture containing pollen grains were placed on a microscopic glass, covered with a cover glass, and examined under a microscope.

## Supplementary Information

Supplementary Information.
