# Supplementary figures and images for: Longevity and germination of Juniperus communis L. pollen after storage
Source: Sci Rep. 2021 Jun 17;11:12755. doi: 10.1038/s41598-021-90942-9 (PMC8211694; doi:10.1038/s41598-021-90942-9)

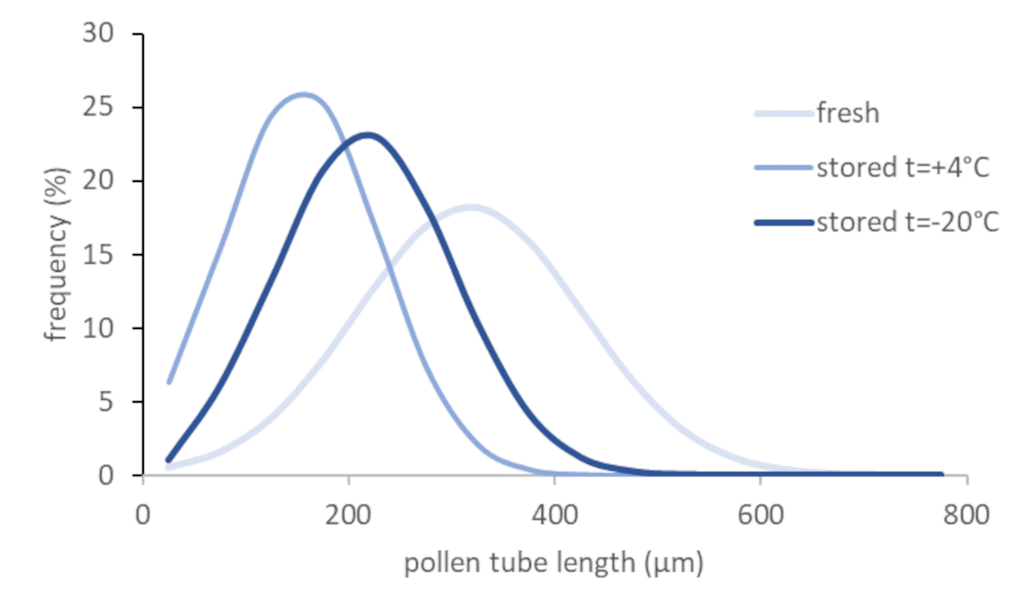


Fig. S1

**Fig. S1** Frequency distribution of pollen tube lengths for individual treatments

Supplement: Supplementary file 1 — Supplementary Information. [file 41598_2021_90942_MOESM1_ESM.docx]
